# Oral manifestations of iron imbalance

**DOI:** 10.3389/fnut.2023.1272902

**Published:** 2023-10-13

**Authors:** Uwitonze Anne Marie, Julienne Murererehe, Mahum Rehman, Mythri Chittilla, Peace Uwambaye, Mohammed S. Razzaque

**Affiliations:** ^1^Department of Preventive and Community Dentistry, School of Dentistry, University of Rwanda College of Medicine and Health Sciences, Kigali, Rwanda; ^2^Department of Pathology, Lake Erie College of Osteopathic Medicine, Erie, PA, United States

**Keywords:** iron deficiency, oral health, iron overload, periodontal diseases, oral malignancy

## Introduction

Iron is the most abundant essential trace element in the human body. Iron exists in two biologically relevant states: the reduced ferrous form (Fe^2+^) and the oxidized ferric form (Fe^3+^). Body iron content is approximately 4.0 gm in men and 3.5 gm in women (1). The functional importance of iron in acting as a cofactor, required for the activity of many enzymes and molecules, lies in its ability to undergo redox cycling between its two predominant oxidation readily states, Fe^3+^ and Fe^2+^ (2). In humans, iron is incorporated into proteins. Iron-containing proteins are required for cellular and organismal functions such as oxygen transportation, nucleic acid replication and repair, mitochondrial respiration, host defense and cell signaling, and intermediary and xenobiotic metabolism (2–5). Iron deficiency and associated anemia are serious public health problems in developed and developing countries (6–9). These iron related diseases can exist as multiple comorbidities with chronic inflammatory diseases such as chronic heart failure, chronic kidney disease, and inflammatory bowel diseases. Despite clinical relevance, unfortunately, its treatment is often overlooked (10). Timely detection and treatment of iron imbalance are crucial because iron is essential for the functionality of all organs. Iron deficiency has been estimated to affect 37–61% of patients with chronic heart failure, 24–85% of patients with chronic kidney disease, and 10–90% of patients with inflammatory bowel disease (10).

On the other hand, iron excess is also harmful to health, which fuels oxidative damage and organ dysfunctions, and one of the contributing factors that leading to cardiomyopathy, diabetes mellitus, liver cirrhosis, and other endocrinopathies (11, 12). Since both iron deficiency and iron excess are associated with numerous adverse health consequences in many organ systems, it is imperative to maintain optimal iron levels in the body. In the healthy state, a required amount of iron is absorbed from the duodenum, and the remaining unabsorbed portion is excreted through feces.

The daily body iron requirements are different based on the age and gender of the individual. The recommended daily allowance has been estimated for adult men above 18 years as 10 mg, while for adult females above 18 years, as about 18 mg. Pregnant women need a higher amount as the mother's body transfers around 15% of absorbed iron to the fetus via the placenta (13). Vegetarians need twice as much iron as non-vegetarians because the human body does not absorb non-heme iron directly from plants. Yet, it can readily absorb the heme iron from meat, poultry, or seafood (14). Dietary sources of heme iron include liver, meat, poultry, and fish, while non-heme iron is provided by cereals, green leafy vegetables, legumes, nuts, oilseeds, and dried fruits (15). The upper limit of iron uptake is around 40–45 mg. This article will briefly discuss the current understanding of iron homeostasis and elaborate on the importance of maintaining iron balance for optimal oral function.

## Iron homeostasis

The body absorbs about 2 mg of iron daily in the duodenum and upper jejunum. The absorption rate of iron in the small intestine is approximately 25–30% (16). This absorption is balanced by iron losses caused by skin desquamation, sloughing of intestinal epithelial cells, and blood loss. In the diet, both heme and non-heme iron forms are present. Most dietary non-heme iron is in the form of Fe^3+^, and it needs to be reduced to Fe^2+^ form to be absorbed (17). The process of being reduced is achieved by the actions of the membrane-bound ferric reductase duodenal cytochrome B (DCYTB or CYBRD1), expressed at the apical brush border membrane of intestinal epithelial cells. Iron absorption is assisted by ascorbic acid, the acidic microenvironment, and the H+ gradient generated by the enteric brush border Na+/H+ exchangers (18, 19). Ascorbic acid is the most potent enhancer of non-heme iron absorption. Other dietary factors, including citric acid and other organic acids, alcohol, and carotenes, enhance non-hemochromatosis absorption. Meat also plays a role in absorbing non-heme iron by stimulating gastric acid production. However, non-heme iron absorption is inhibited by phytic acids (17); iron absorption is inhibited even from tannins and oxalic acid.

After absorption, Fe^2+^ iron is transported across the apical border of enterocytes by the divalent metal transporter 1 (*DMT1*) and across the basolateral surfaces of intestinal enterocytes by the iron exporter, ferroportin, to the systemic circulation (20). Ferroportin is needed to transport Fe^2+^ from other cell types like enterocytes, macrophages, and hepatocytes into plasma (21). To release Fe^2+^ from the biological stores, caeruloplasmin or hephaestin, a copper-containing ferroxidase enzyme assists ferroportin. Since both iron deficiency and iron overload are detrimental, organisms have developed regulatory mechanisms for balancing iron within safe limits (2, 22).

In humans, like in all mammals, there is no mechanism for excreting excessive iron, which is why iron homeostasis is regulated at the place of absorption, utilization, and recycling (20). Iron is only lost from the body through skin desquamation and sweat, urines, gastrointestinal secretions, hair, and menstrual bleeding in premenopausal women. The amount of iron lost through the above-mentioned sites is minimal, approximately 1 mg/day in males and slightly more in child-bearing women due to menstruation, pregnancy, and lactation (13).

Biologically available iron is scarce, which is why humans efficiently conserve and recycle iron (22). Even though all cells may import, export, or store iron, some have specific functions, like erythroblasts for iron uptake, macrophages and enterocytes for iron export, and hepatocytes for iron storage (23). Iron taken up by enterocytes can be used in three ways: (1) it can be used directly in cells for intrinsic cellular metabolic processes; (2) it can be stored in the enterocytes in an inert form, limiting the production of damaging reactive redox species; (3) it can also be exported in the systemic circulation across the basolateral membranes (24).

The mechanism regulating systemic iron takes place in the liver involving hepcidin and ferroportin, which work together to control the flow of iron from cells to the systemic circulation. Identifying hepcidin has helped to understand how the liver is the central regulator of iron homeostasis and why liver deregulation leads to iron disorders (18, 24). Hepcidin is an iron-regulating hormone that controls iron absorption by binding to ferroportin (25). Hepcidin is not an enhancer of iron absorption. On the contrary, it functions as the primary inhibitor of iron transport from cells, either enterocytes, macrophages, or hepatocytes, to the bloodstream. When it binds to the extracellular arm of ferroportin, iron is internalized and degraded, preventing it from regression. The blocked iron is finally excreted through the stool when the enterocytes die (26). Therefore, stimulating hepcidin expression reduces iron absorption from the diet and iron release from macrophages and other body stores, while lowering hepcidin levels increases iron availability (27). The expression of hepcidin is regulated by different signals that communicate the body's need for iron. These signals are intensified by iron, erythropoietic drive, and inflammation (27).

**Figure 1:**
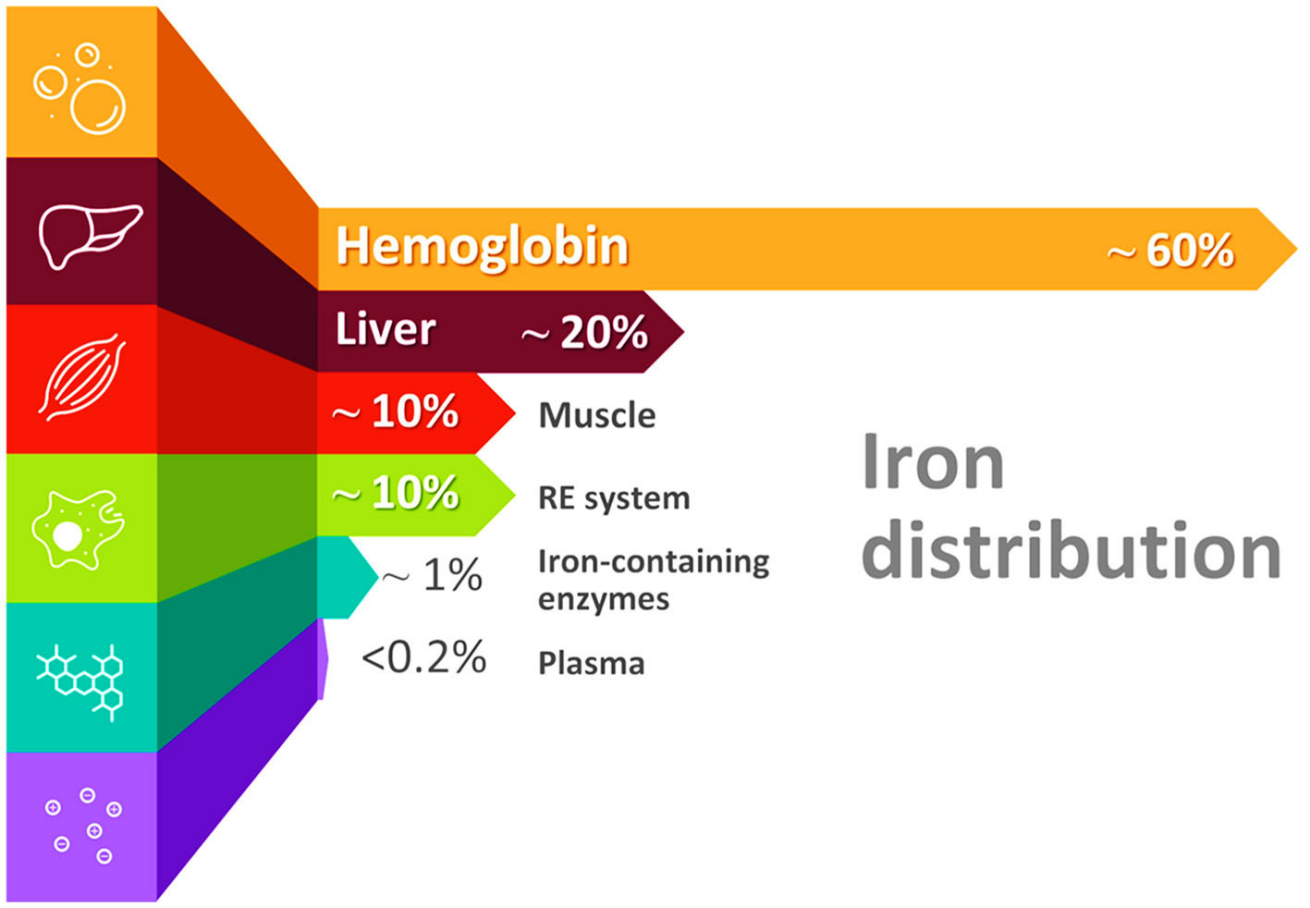
Distribution of iron in the body. Please note that nearly 60% of iron is found in the hemoglobin of red blood cells (erythrocytes) in the form of heme (RE system, reticuloendothelial system).

The majority of iron (60–70%) is in the hemoglobin, 10% is in muscle myoglobin, and the remaining 20–30% are found primarily in iron storage pools located in the liver and the reticuloendothelial (macrophages) system as ferritin and insoluble hemosiderin. Only 1% is incorporated in iron-containing enzymes, and <0.2% of body iron is circulating in the plasma, bound to transferrin (Figure 1) (28). Part of the iron is also contained in the functional hemochromatosis group, a component of the electron transport chain. Only 2–4 mg of iron constitute the circulating pool, and since that amount is small, it must be turned over every few hours to meet the daily iron requirements, which are around 20–25 mg (20).

Iron recycling constitutes a major source of daily iron requirements, 90–95% of daily iron requirements for erythropoiesis (19). That is achieved through macrophages which phagocytes old and damaged red blood cells after 120 days, when their lifespan ends. These red blood cells are lysed, and through the oxygenation of the hemochromatosis component by hemochromatosis oxygenase-1, and iron is released (25). This released iron can then be reused to form new erythrocytes in the bone marrow (19).

Iron entry into the bloodstream is controlled by the iron transporter, transferrin, which acts as a gatekeeper (29). The iron form bound to transferrin is Fe^3+^, meaning that Fe^2+^ must be oxidized to Fe^3+^ for transportation (30). Under normal conditions, transferrin-bound iron is the main form of iron in circulating blood. When the present iron exceeds the carrying capacity of transferrin, a non-transferrin-bound iron form can circulate, including highly reactive labile plasma iron (LPI), and reach organs such as the liver, the heart, and the pancreas, leading to organ damage (31).

## Causes of iron deficiency

The causes of iron deficiency differ according to age, gender, and socioeconomic status. Iron deficiency may result from insufficient iron intake, decreased absorption, or blood loss, particularly in the elderly. Low dietary intake increases systemic requirements during pregnancy, and parasitic infestations, especially in developing countries, can lead to iron deficiency (22). The highest prevalence rates of iron deficiency and anemia occur in low-income countries due to the combination of a poor plant-based diet and chronic infections, which limit iron uptake (26, 30). However, iron deficiency is also a concern in developed countries, especially for young women (17). Young children, women with heavy menstrual cycles, frequent blood donors, adolescent girls, strict vegetarians, and the elderly are vulnerable to iron deficiency. Iron deficiency can also be genetic or metabolic. Genetic iron deficiency is linked to mutations of genes involved in the transport and metabolism of iron. Mutations in STEP3 and DMT1 lead to macrocytic anemia, while mutations in serine 6 (TMPRSS6) gene would lead to Iron Refractory Iron Deficiency Anemia (IRIDA), where there is abnormally elevated levels of serum hepcidin which results in suppression of iron absorption and recycling. It may require intravenous iron, especially when iron demand is high (18, 27). As stated earlier, some causes of iron deficiency are nutritional deficiency, malabsorption, increased iron requirements such as during pregnancy and growth, and increased blood loss (17, 32). Patients with chronic kidney disease are at a greater risk of iron deficiency anemia because they often suffer nutritional deficits and are taking medications that disturb enterocyte iron uptakes, such as phosphate binders and antacids; furthermore, there is an increased iron utilization due to erythropoiesis-stimulating agents, and increased blood loss due to hemodialysis, frequent phlebotomy, and uremic platelet dysfunction (33).

Despite the well characterization of iron deficiency anemia, there is less research with regard to iron deficiency without anemia. Iron deficiency without anemia may cause neurocognitive health issues among adults. Extreme cases associated with blood loss, regular blood donation, consumption of diets with low bioavailability of iron, and challenges arising during pregnancy may increase the risk of iron deficiency in the population. Care should be taken when determining the reference ranges for iron in patients. This is because some physiological changes in the body may cause false changes in iron levels, like how fasting is shown to create diurnal variations in serum iron levels (34). Other markers apart from hemoglobin must be used in laboratory assessment; hemoglobin has low specificity and sensitivity. The testing is necessary because significant changes in the levels of iron may adversely affect the body's physiology.

## Iron deficiency in dental caries and periodontal diseases

The resistance of teeth to dental caries depends on the hardness of their enamel. Previous studies have demonstrated that iron is one of the trace elements found in the hydroxyapatite of the enamel, contributing to a low risk of dental caries. In the study, 38 human teeth were extracted to compare the concentration of trace elements in enamel to the rest of the body. Using inductive coupled plasma-optical emission spectroscopy (ICP-OES), it was found that among the 19 trace elements, the concentration of iron in the enamel was similar to the concentration in other parts of the body (35). Iron deficiency was statistically associated with dental caries (36–38). Iron deficiency can influence the growth of teeth and promote dental caries. A study done in Turkey found that iron deficiency can promote dental caries because it may lead to an early eruption of deciduous teeth with a negative impact on the enamel. An early eruption can predispose to dental caries because the longer the period a tooth is in the oral cavity, the more the risk for dental caries. The same researchers also stated that premature tooth eruption could lead to malocclusion, hindering proper oral hygiene and promoting periodontal diseases (39). Another study performed in Canada, aiming to determine if there is a relationship between severe early childhood caries and iron status, found that children with severe early childhood caries have significantly low hemoglobin levels, low ferritin levels, and low iron levels. The adjusted odds of children with severe early childhood caries having low ferritin levels were almost double compared to children without dental caries. Researchers proposed pre-assessment of children with severe early childhood caries before surgery, including iron and hemoglobin levels (40). Similar results were obtained in India, where severe early childhood caries were found to be statistically associated with low serum ferritin and low hemoglobin levels. Some theories are put forward, such as how iron deficiency reduces saliva buffering capacity of salivary glands. Summarizing the study results, iron has a cariostatic effect on dental caries. Experimental studies confirmed this effect; when iron is added to cariogenic foods, it can reduce the incidence of dental caries in animals or humans. Such effects were attributed iron's ability to strongly inhibit the glucosetransferrase enzyme (GTF) and remineralize enamel. In addition, studies have shown that iron is able to cover the enamel surface with protective layers (41, 42). It was found that iron ions precipitations on the enamel create an acid-resistant slim coating that contains gels and crystals of hydrated iron oxide, absorbing salivary calcium and phosphates thereby nucleating the formation of appatites and the replacement of minerals during the acid phase of the carious process.

Individuals who are genetically predisposed to low levels of iron also develop oral complications. Patients suffering from β-thalassemia major have high dental caries and gingival inflammation (43). Oral health was assessed in children with β-thalassemia major, an autosomal recessive disorder depicted by severe anemia and lifelong blood transfusion, in relation to their serum ferritin level (43). Early Childhood Oral Health Impact Scale (ECOHIS) was used to measure oral health-related quality of life (OHRQoL). Individuals with serum ferritin levels below or above 2,000 ng/mL (*p* > 0.05) scores were high for dental plaque and gingival bleeding in β-thalassemia major patients (43). The mean was 3.2 teeth for decayed, missing, and filled tooth surfaces in BTM patients. Responses from all participants to 13 questions of the ECOHIS indicated 53% of the individuals with serum ferritin levels ≥2,000 ng/mL usually experienced dental pain compared to those with serum ferritin levels <2,000 ng/mL (*P* > 0.05) (43). It was also found that 42% of the serum ferritin level ≥2,000 ng/mL group usually had trouble drinking hot or cold beverages compared to 25% of the individuals with serum ferritin levels <2,000 ng/mL (*p* > 0.05) (43). Please note that “below or above 2,000 ng/mL” refers to two experimental groups, not a numerical reference range. This study and others confirmed that β-thalassemia major patients are prone to dental caries. Another study was conducted, which compared the salivary flow rate, buffering capacity, resting pH, and dental caries in β thalassaemic children with healthy children (44). This study measured salivary parameters using a saliva check buffer kit, and dental caries were recorded using WHO criteria 1997. It was found that there was a positive correlation between dental caries and salivary parameters in thalassaemic children, which may be due to frequent blood transfusions, immune deficiency, or poor oral hygiene.

Iron deficiency anemia is also associated with severe periodontitis. Iron is essential for most microbes, including oral microbiota (45). Since the mouth harbors more than 700 species of bacteria existing in the form of a biofilm which maintains ecological balance with the host in a physiological state, it is imperative to prevent the disruption of that balance, which could lead to oral infectious diseases such as dental caries, periodontal diseases, periapical periodontitis and pericoronitis (46). *In vitro* and *in vivo* previous studies have demonstrated that iron deficiency is able to shift the ecological balance to specific pathogenic bacteria (40, 45). Concerning the association between iron deficiency and periodontal disease, a case was reported where eight teeth were extracted. The patient presented generalized bone loss and teeth mobility while the oral hygiene was not poor. There was neither plaque nor calculus, and deep medical investigations revealed severe anemia. When the anemia was corrected, oral and general health became stable (47).

## Iron deficiency in oral premalignant or malignant conditions

Adequate oxygen transport to all tissues, including oral mucosa, periodontium, and teeth, is essential for good oral health. Oxygen is transported to the tissues by hemoglobin, which is a combination of hemochromatosis and globin protein. The lack of iron can lead to oral infection and bacteria accumulation due to inadequate oxygen. Iron deficiency anemia is the most common manifestation of low serum levels of iron, and it can manifest in the oral cavity by angular cheilitis, atrophic glossitis, generalized oral mucosal atrophy, candida infections, recurrent aphthous ulcers, pallor, and stomatitis (48). Atrophic glossitis is manifested as the atrophy of fungiform and filiform papilli, which progresses to the lateral borders and the dorsum of the tongue. The tongue may show bald patches or be red, shiny, and smooth; it feels sore, burning and tender (49). On the other side, excess iron in drinking water can cause extrinsic stains (50).

**Figure 2:**
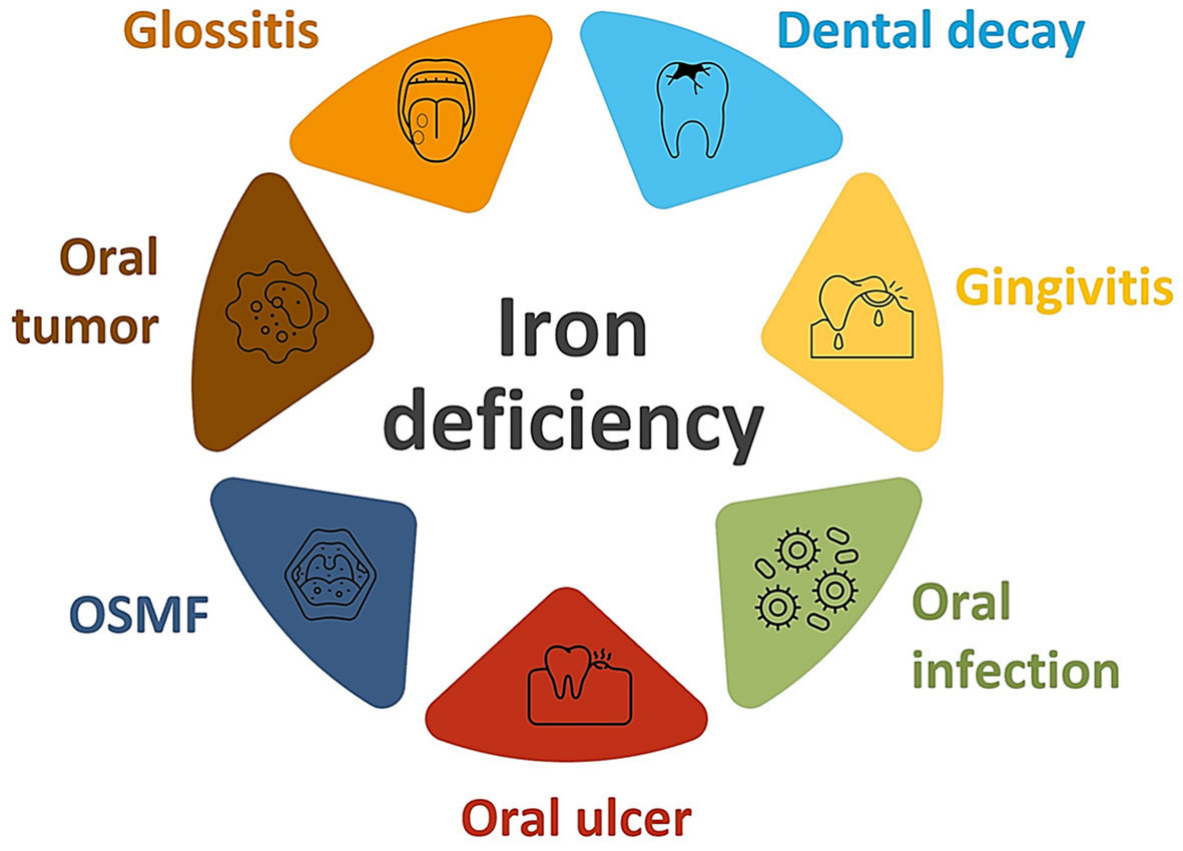
Iron deficiency and oral lesions (OSMF, oral submucous fibrosis).

Iron deficiency has also been associated with oral premalignant lesions (Figure 2). In fact, a significant decrease in serum iron concentration with elevated total iron binding capacity has been found in patients suffering from oral submucous fibrosis (32, 51, 52). It was found that iron deficiency in oral tissues results in decreased vascularity which enhances the percolation of arecoline, which is a byproduct of the areca nut. Areca nut, commonly referred to as betel nut, is found in south and southeast Asia and contains carcinogens, like arecoline. When that percolation increases, arecoline damages the oral mucosa by enhancing fibroblastic proliferation and collagen formation. Battacharya and colleagues reported a case where iron deficiency primarily resulted in the development of oral submucous fibrosis, which was successfully treated by iron supplements and antioxidants (32). Low serum iron levels have also been found in patients suffering from oral leukoplakia. Furthermore, iron deficiency has been associated with Plummer-Vinson syndrome, also known as Paterson-Brown-Kelly syndrome or sideropenic dysphagia; this dysphagia has the potential to transform into malignancy. A relationship was established between iron deficiency and esophageal adenocarcinoma (53).

Combined with other trace elements and various micronutrients, iron has been used to effectively treat oral diseases such as oral leukoplakia, oral submucous fibrosis, and oral cancer. Furthermore, it was found that hemochromatosis might be used as a follow-up tool for the progression of head and neck carcinomas because it has been observed that serum levels are elevated, whereas iron concentrations are decreased with the tumor progression (32).

## Management of iron deficiency

Iron deficiency can be treated by dietary change, drugs, and surgery, but dietary uptake or diversification is the most sustainable approach. The treatment depends on the cause and severity. Oral iron is the first line, inexpensive, safe, and convenient treatment (54). However, oral iron treatment can be challenging for patients with gastrointestinal bleeding due to gastrointestinal side effects, malabsorption, or requirements of higher supplementation doses which aggravate side effects (55). Furthermore, a large amount of dietary iron can induce the enterocytes to develop a “mucosal block”, reducing iron uptake for several days, even in the presence of systemic iron deficiency. When a dietary approach does not correct the problem, iron supplements are provided, preferably with vitamin A, and in very severe cases, blood transfusions and parenteral iron infusions are given (56, 57). Strategies to control deficiencies include dietary diversification, food fortification, and iron supplementation.

Iron deficiency anemia should start by treating the underlying cause and oral iron supplementation using ferrous sulfate. Rises in hemoglobin levels are often seen in 14 days, but iron supplementation is needed for at least 3 months to replenish tissue iron stores. It should be maintained for at least 1 month, even after the hemoglobin reaches normal levels (58). In some cases, intravenous iron administration is needed, like when the patient does not tolerate oral iron supplementation or when he has malabsorption like in celiac disease, post-gastrectomy, or achlorhydria, or when the losses are too high for oral therapy (59, 60). However, the long-term effects of intravenous correction of iron deficiency in inflammation should be monitored closely because that can lead to macrophage iron accumulation. A new proposed therapeutic opportunity is to manipulate the hepcidin pathway by blocking its production or function, especially when other treatments are unsatisfactory (27, 61).

## Iron overload and oral health

**Figure 3:**
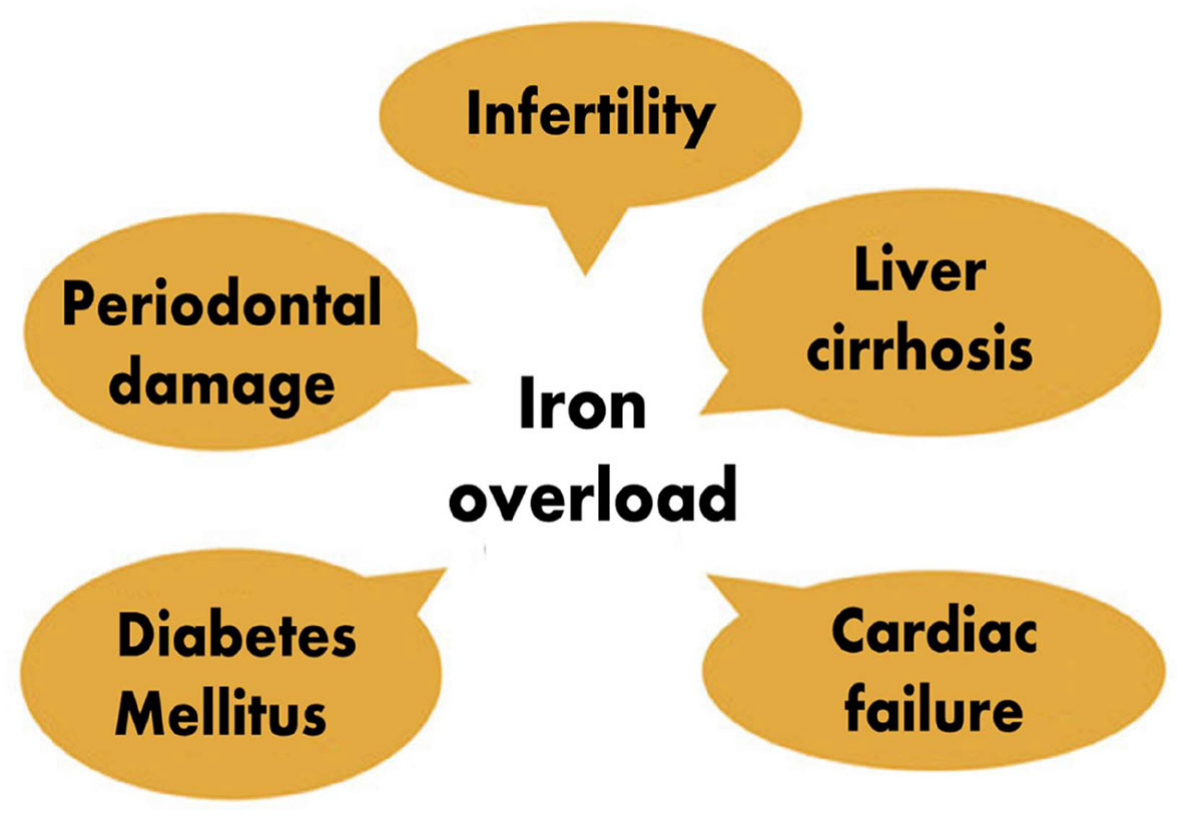
Partial list of effects of iron overload (11, 12).

The two primary sources of iron overload are continuous transfusions and increased intestinal iron absorption. Thalassemia is a chronic autosomal recessive condition which is characterized by severe anemia and necessitates frequent blood transfusions. These patients are at risk of developing transfusion-related iron overload (23). As stated above, iron deficiency has been associated with severe periodontitis. However, studies also documented that iron overload is equally harmful to the periodontium. An increased transferrin saturation (TSAT) level exceeding 45% has been found to promote severe periodontitis. Patients with TSAT above 45% were found to be at 4 to 5 times higher risk of developing severe periodontitis due to the fact that iron is an essential growth-promoting factor of *porphyromonas gingivalis* and that it can act as its virulence factor, especially in patients with genetic hemochromatosis (62). There is an association between *porphyromonas, treponema*, and TSAT; increased TSAT is associated with oral dysbiosis characterized by the elevation proportions of periopathogens which may participate in periodontium destruction. In addition, non-transferrin-bound iron-associated cellular toxicity has also been incriminated. Patients with genetic hemochromatosis have hepcidin deficiency, which can promote low iron content in macrophages, destroying periodontal tissues (46). Animal studies have revealed that iron overload leads to decreased inter-radicular bone volume by its inhibiting effect on bone formation. That explains why iron overload leads to loss of alveolar bone, worsening periodontal disease (47). However, it has also been observed that the invasion of periodontal pathogens can cause iron overload. Researchers found that chronic periodontitis was a risk factor for increased serum ferritin, increased serum hepcidin, and decreased hepcidin/ferritin ratio. They hypothesized that chronic periodontitis might be an independent risk factor for iron overload and inadequate hepcidin production (63). This becomes a vicious circle, iron overload worsening periodontitis and periodontal pathogens causing iron overload with all its complications of systemic diseases. Patients suffering from thalassemia major have a maxillary enlargement which causes facial and oral symptoms such as teeth protrusion, spacing, occlusal deep bite, open bite and different degrees of malocclusion which then promotes dental caries and periodontal diseases, especially because these patients neglect their oral hygiene and the modification of their saliva biochemical constituents (43). In addition to oral lesions, iron overload can induce wide-range of organ damages, including cardiac and hepatic leions (11, 12) (Figure 3).

Iron overload has been associated with black stains on teeth surfaces in people with iron metabolism disorders. The primary cause of this pathology of black stains is the accumulation of iron in tissues and secretions, together with chromogenic bacteria. In case of iron metabolism disorders, hydrogen sulfide synthesized by oral bacteria reacts with iron in saliva and forms black precipitates consisting of ferric sulfide. These precipitates bind to the surface of the teeth, forming an unsightly stria that follows the gingiva contour. These can be removed by electronic scaler and polishing pastes, but the black stains usually recur shortly (64).

## Conclusion

Since the oral cavity has been called the mirror of the human body because many systemic diseases have relatively early oral manifestations, it is imperative that iron deficiency and associated anemia be examined in children with early childhood caries for early treatment, thus helping to prevent more harmful complications later in life. Dental practitioners should have the ability to recognize early warning signs of low iron levels, such as early childhood caries in order to allow patients to receive necessary interventions before the longstanding effects of iron deficiency are manifested. Dental professionals should also be conversant with oral manifestations of iron overload to detect them early and refer them for treatment to avoid severe complications. Finally, maintaining optimal iron balance, along with other minerals throughout life is essential for normal bodily functions, including oral ones (65–74).

## Author contributions

UA: Writing—original draft. JM: Writing—original draft. MR: Writing—review and editing. MC: Writing—review and editing. PU: Writing—original draft. MSR: Writing—review and editing, Conceptualization, Supervision.
